# Preclinical investigations towards the first spacer gel application in prostate cancer treatment during particle therapy at HIT

**DOI:** 10.1186/1748-717X-8-134

**Published:** 2013-06-06

**Authors:** Antoni Ruciński, Julia Bauer, Patrick Campbell, Stephan Brons, Daniel Unholtz, Gregor Habl, Klaus Herfarth, Jürgen Debus, Christoph Bert, Katia Parodi, Oliver Jäkel, Thomas Haberer

**Affiliations:** 1 Heidelberg Ion Beam Therapy Center and Department of Radiation Oncology, Heidelberg University Clinic, Im Neuenheimer Feld 400, Heidelberg, 69120, Germany; 2 Augmenix Inc., Waltham, MA, USA; 3 Biophysics division, GSI Helmholtzzentrum für Schwerionenforschung GmbH, Planckstraße 1, Darmstadt, Germany

**Keywords:** Radiation therapy, Particle therapy, Prostate cancer, Spacer gel

## Abstract

**Background:**

The application of spacer gel represents a promising approach to reliably spare the rectal frontal wall during particle therapy (IJROBP 76:1251-1258, 2010). In order to qualify the spacer gel for the clinical use in particle therapy, a variety of measurements were performed in order to ensure the biological compatibility of the gel, its physical stability during and after the irradiation, and a proper definition of the gel in terms of the Hounsfield Unit (HU) values for the treatment planning system. The potential for the use of the spacer gel for particle therapy monitoring with off-line Positron Emission Tomography (PET) was also investigated.

**Results:**

The spacer gel implanted to the prostate patient in direct neighbourhood to the clinical target volume does not interfere with the particle therapy treatment planning procedure applied at Heidelberg Ion Beam Therapy Centre (HIT). The performed measurements show that Bragg-peak position of the particles can be properly predicted on the basis of computed tomography imaging with the treatment planning system used at HIT (measured water equivalent path length of 1.011 ±0.011 (2*σ*), measured Hounsfield Unit of 28.9 ±6.1 (2*σ*)). The spacer gel samples remain physically unchanged after irradiation with a dose exceeding the therapeutic dose level. The independently measured Bragg-Peak position does not change within the time interval of 10 weeks.

**Conclusions:**

As a result of the presented experiments, the first clinical application of spacer gel implant during prostate cancer treatment with carbon ions and protons was possible at HIT in 2012. The reported pre-clinical investigations demonstrate that use of spacer gel is safe in particle therapy in presence of therapy target motion and patient positioning induced particle range variations. The spacer gel injected between prostate and rectum enlarge the distance between both organs, which is expected to clinically significantly decrease the undesirable exposure of the most critical organ at risk, i.e. rectal frontal wall. Further research on the composition of spacer gel material might lead to additional clinical benefits by validation of particle therapy of prostate via post-therapeutic PET-imaging or by patient positioning based on the gel as a radio-opaque marker.

## Background

Prostate cancer is the predominant cancer in men in developed countries. Clinical trials for intensity modulated radiation therapy (IMRT) demonstrated that the increase of the target dose for prostate cancer enables better tumour control [1,2]. In order to guarantee a successful therapy, the clinical target volume must be entirely covered by the prescribed dose during all treatment fractions. In clinical practice, limitations in the accuracy of the treatment delivery are often dealt with by enlarging safety margins around the target volume. This margin extension strongly depends on the applied radiation technique, availability of image guidance and selection of the patient positioning protocol, inter- and intrafractional target motion, and dose-exposure limits of neighbouring organs at risk.

Carbon ion therapy is expected to be an efficient method for treating prostate cancer due to its high conformity and radiobiological effectiveness [3,4]. The high conformity in particle therapy is challenged by organ motion that may strongly affect the target dose distribution. A variety of studies have already reported the impact of intra- and interfractional prostate motion on the precision of photon radiotherapy, e.g. [5-7]. Due to the finite range of the ions in tissue, further deviations in the target dose may occur as a result of variations in material density distribution over the particle path mainly induced by daily target motion and patient positioning [8]. For pre-treatment patient positioning the Heidelberg Ion Beam Therapy Centre, Heidelberg, Germany (HIT) offers image guided radiation therapy based on a digital X-ray system. This implies the application of a bony anatomy registration protocol for the positioning of prostate cancer patients. The clinical target volume (CTV), i.e. prostate, is located between two organs at risk (OAR): rectum and bladder. Due to the day-to-day target motion, the rectal frontal wall may shift into the high dose region of the field previously defined for the target volume. This leads to unquantified exposure of rectum and may result in rectal toxicity [6]. The tendency to apply a hypofractionated prostate treatment protocol emphasises this problem [9,10]. For these reasons the application of soft tissue like spacer gel (SpaceOAR™System, Augmenix Inc., Waltham, MA, US) enlarging the distance between radiotherapy target and organ at risk [11], not inducing particle range variations, represents a promising approach to reliably spare the rectal frontal wall during particle therapy. At HIT first prostate patients were treated with scanned carbon ion and proton beams using therapy. At HIT first prostate patients were treated with scanned carbon ion and proton beams using spacer gel in In order to qualify the spacer gel for the clinical use, a variety of measurements is required to guarantee the biological compatibility of the gel, its physical and chemical stability during and after the irradiation, and a proper definition of the gel in terms of Hounsfield Unit (HU) values for the treatment planning system (TPS). The potential for the use of the spacer gel for particle therapy monitoring with offline Positron Emission Tomography (PET) is also an issue of interest. This paper reports on the set of measurements performed in collaboration of Augmenix Inc., Waltham, MA, US, and HIT. The presented results and the clinical experience of Heidelberg University Clinic in application of spacer gel in photon radiotherapy are the basis for the first application of spacer gel during prostate therapy with carbon ions and protons at HIT.

### Bio-compatibility of the spacer gel implant

The SpaceOAR hydrogel consists primarily of water and crosslinked polyethylene glycol (PEG). PEG is widely used in the pharmaceutical, cosmetic and medical device industries due to its low toxicity and lack of immunogenicity. In use, the SpaceOAR hydrogel is applied into the potential space between Dennonvilliers’ Fascia and the frontal rectal wall under transrectal ultrasound guidance. Following placement of an 18G needle, the liquid hydrogel precursors are injected, expanding the space. Within 10 seconds ester linkages on the PEG crosslink with amine endgroups on trilysine, causing the hydrogel to polymerize (solidify) without a measurable rise in temperature. The ester at each PEG-trilysine linkage is water sensitive, allowing the network to hydrolyze and liquefy over time. The kinetics of this hydrolysis are such that the hydrogel remains in place for three months, after which it liquefies, is absorbed, and cleared via renal filtration within six months of implantation.

Prior to clinical evaluation the SpaceOAR hydrogel underwent extensive biocompatibility testing per ISO 10993 (International Organization for Standardization, http://www.iso.org/) performed at Augmenix. To evaluate the potential effects of irradiation on hydrogel compatibility, tests were performed on gels with exposure to 150 Gy photon irradiation, and on gels with no irradiation. Augmenix reports that, as anticipated, the gel was found to be non-cytotoxic, non-sensitizing, non-genotoxic, and with no signs of local or systemic toxicity, even at high doses. In a 16-week intramuscular test on the rabbit, the hydrogel was rated as a non-irritant, the same as the negative control high density polyethylene.

### Treatment planning

Predicting the energy loss of charged particles and their respective range in the patient’s body (physically described by the position of the Bragg-Peak) is one of the objectives of radiotherapy planning with particles [12]. The treatment plan is calculated starting from Computed Tomography (CT) images containing information about the electron density distribution in the human body given in HU values. On the basis of the spatial distribution of HU values within the CT image the particle range in material is predicted by a semiempirical scaling to the particle range in water by means of the WEPL (water equivalent path length) calibration. The relation of the HU values to WEPL (defined as Hounsfield unit Look Up Tables - HLUT) is the basis for treatment plan optimisation and dose determination. The piecewise linear function HLUT is empirically estimated by measuring HU values and particle ranges (WEPL) of tissue-equivalent materials using the stoichiometric method [13]. For this reason the safe application of the non-tissue implant of spacer gel in the area of the planning target volume (PTV) requires that the range of the particles crossing the gel during the irradiation is the same as their expected range estimated by the TPS (on the basis of CT scan and HLUT). The HU value of the spacer gel sample was determined at the CT scanner commissioned for treatment planning at HIT. The WEPL of the spacer gel was experimentally estimated with water absorber of varying thickness [14] in measurements at different points in time and in an additional measurement after exposure to the high dose. These verifications are crucial to ensure that the spacer gel implant holds its original properties as assumed by the treatment planning over the whole therapy course. On the basis of these investigations the compatibility of the spacer gel stopping properties with the HLUT used for patient treatment plan optimisation at HIT was validated.

### Application for marking purposes

Beyond its main task, i.e. the spacer gel being implanted in the direct proximity of the actual treatment volume but not being involved in biological processes, it might also be exploited for marking purposes, for example in PET-based treatment monitoring or as a marker for patient pre-treatment positioning using X-ray imaging.

At HIT, post-therapeutic PET measurements are performed for in-vivo treatment verification [15]. Within the irradiated volume, *β*^+^ emitting isotopes are produced in inelastic fragmentation reactions between the beam particles and the tissue. The induced activity is measured offline with a commercial PET/CT scanner subsequent to the treatment fraction with a few minutes of delay due to patient transportation to the scanner (located next door to the treatment rooms) and repositioning. Correlating the measured activity distribution to an expectation calculated under the assumption of a correct treatment delivery allows verifying the beam position and the beam range in the target volume in suitable tissue regions [16,17]. [16,17]. Owing to the human tissue composition and the half-life of the produced isotopes, dominated by ^11^C (*T*_1/2_ ≈ 1222 *s*), with smaller contributions of ^15^O (*T*_1/2_ ≈ 120 *s*), ^13^N (*T*_1/2_ ≈ 598 *s*) and ^38^K (*T*_1/2_ ≈ 458 *s*).

Due to the tissue-equivalence of the known chemical composition of the spacer gel (c.f., Section Spacer gel samples) it is expected to be activated in a similar way as the surrounding patient tissue. However, in contrast to the natural tissue, where the induced activity is washed out by physiological processes, which reduces significantly the measurable signal level, the isotopes generated in the spacer gel are locally confined to the production spot. Due to this fact, the offline PET/CT-based monitoring of the prostate patients potentially enables the usage of the activated spacer gel as an in-vivo marker for inter-fractional positioning verification. The level of activity induced in the irradiation of the spacer gel is expected to be comparatively low, since ^15^O is the most abundantly generated isotope, which, however, decays to a large extent during the time delay between end of irradiation and start of PET measurement. Nevertheless, a study of the isotope separation in experimental data provides valuable information to validate the chemical gel composition and to investigate the benefit of the gel as a potential activity marker based on realistic patient treatment scenarios. In the present study we report the pre-clinical phantom studies, conducted at HIT to evaluate the feasibility of this particular spacer gel application.

## Methods

### Spacer gel samples

The spacer gel injected to the patient polymerises in seconds and creates a volume of about 10 ml. The composition of the gel designed by Augmenix is 90% water and 10% polyethylene glycol (PEG, chemical composition: C_2_H_6_O_2_) yielding only a low contrast against the surrounding tissue on CT images. For the range measurement the gel implant was injected into a plexiglass (PMMA) vial manufactured for the experiment and closed to the air by a precisely fitted cap. The vial creates the cylindrical space for the spacer gel sample of 20 mm diameter and 32 mm length which result in 10.05 ml volume. The vial made of PMMA was designed to be watertight after the caps are locked. In order to guarantee exact positioning of the vial for the experiment in a dedicated vial holder, the vial was manufactured with an overall tolerance of 0.05 mm (including axial tolerance of outer surface, planarity of the vial cap surfaces involved in beam range measurement). Assuming a WEPL of one millimetre PMMA material of 1.165 mmH_2_O [18] the WEPL of two caps (2 x 6.85 mm) is 15.96 mmH_2_O.

Other vials, empty and filled with distilled water, were used for reference measurements. The same gel sample was also adapted for the high dose exposure test described in Section High dose irradiation.

In order to assess the effects of irradiation on SpaceOAR hydrogel, further samples were dedicated for functional and chemical tests performed by Augmenix before and after an exposure to 150 Gy photon irradiation, and to 33 Gy carbon ion and 91 Gy proton irradiation. The design of the samples used in these tests does not impact the quality of results; thus it is not described here.

### Particle range measurement

**Figure 1:**
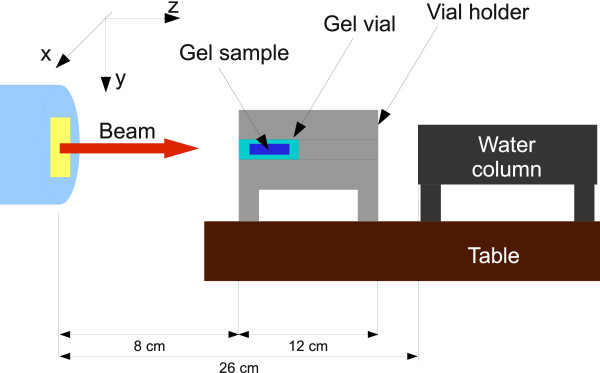
**Schematic view on the spacer-gel range measurement setup.** Using the three plane laser system indicating the beam line and the isocentre a vial containing the gel sample (inserted in the vial holder) was positioned axisymmetric to the horizontal beam line. In order to minimise effects of lateral scattering of particles the distance from the beam exit to the water column was minimised.

Measurements of the particle range in the spacer gel samples were performed with a PEAKFINDER Water Column (PTW, Freiburg, Germany), a water absorber of varying thickness (water column installed between two parallel-plane ionisation chambers) commonly used for experimental determination of the WEPL of materials. The samples were positioned upstream to a water absorber as it is schematically drawn in Figure 1. Within one measurement session the cylindrical vial containing gel sample was rotated axially to the beam line in the vial holder causing penetration of the sample at slightly different positions. The position of the Bragg-Peak was estimated by varying the water column thickness and measuring the ratio of the charge deposited in the ionisation chambers as described in [18]. The range measurements were performed with carbon ion pencil beams with an energy of 200 MeV/u, a Gaussian focus profile of 5.1 mm full width at half maximum (FWHM), and an intensity of 5.0·10^6^ particles/s.

In order to investigate the stability of the particle penetration depth in the gel sample over time (treatment course), the measurement session was repeated three times within 10 weeks. A measurement session covered reference measurements and measurement series of the vial filled with spacer gel. The reference measurements contained: (i) range measurement without any absorber, (ii) in presence of an empty holder, (iii) in presence of a holder with an empty vial, and (iv) with the vial filled with water. In order to verify the quality of the reference measurements, normalisation was performed with respect to the calculated values. It was expected that after normalisation the reference measurements obtained during various sessions give maximal error not larger than the error of single acquisitions with water column (0.2 mmH_2_O). Putting the spacer gel sample in the field of the beam causes a water equivalent shift of the reference Bragg-Peak position, which is the basis for calculating the absolute WEPL of the spacer gel:

(1) $$\text{WEP}L_{\text{gel}}=1+\frac{\left(\text{via}l_{w}-\text{via}l_{g}\right)}{d}$$

whereas: 

*WEPL*_*gel*_ - calculated water equivalent path length for the gel sample,

*vial*_*w*_ - Bragg-Peak position measured for the vial filled with water,

*vial*_*g*_ - Bragg-Peak position measured for the vial filled with spacer gel,

*d* - thickness of the tested material in mm (without top and bottom vial cap).

Since WEPL of the spacer gel was estimated in a relative way with respect to the reference measurement performed with vial filled with water (*vial*_*w*_), the normalisation procedure has a verification purpose and does not influence the calculated *WEPL*_*gel*_.

The HU value of the spacer gel sample was estimated using CT unit of the Siemens Biograph mCT scanner [19] (later called HIT-PET/CT) being commissioned for treatment planning at HIT. The scan was performed using the body protocol, that is also applied during treatment planning of prostate patients at HIT: body reconstruction kernel (B40s), a tube output voltage of 120 kV, an integrated current of 255 mAs, and a reconstruction diameter of 500 mm. The vial containing the spacer gel used for the range measurement was positioned in the centre of a spherical PMMA phantom with a diameter of 16 cm. The phantom was positioned centrally in the field of view (FOV) of the scanner. The pixel spacing of the obtained images was 0.6055 mm, whereas the slice thickness was 3 mm. The HU value of the gel was determined with Siemens RT planning software package (version VA11A). A circular region of interest covering the spacer gel (15 mm diameter) was drawn on multiple slices and the average HU value including the standard deviation was calculated by the system.

### High dose irradiation

In order to investigate the stability of the spacer gel properties after exposure to a high dose, both the gel sample dedicated for range measurements (after closing particle penetration depth measurement session) and the gel sample provided by Augmenix were exposed to a dose exceeding the dose level that is applied in clinical studies on prostate cancer at HIT. Two irradiation plans were generated with TRiP98 [12] software package (Treatment Planning for Particles, developed at GSI Helmholtz Zentrum fur Schwerionenforschung, Darmstadt, Germany). For both, carbon ions and protons, the RBE weighted dose of 100 Gy (RBE), delivered in one irradiation, was prescribed to the target, whereas the actual clinical protocol prescribes 66 Gy (RBE), delivered in 20 fractions to the clinical target volume.

**Figure 2:**
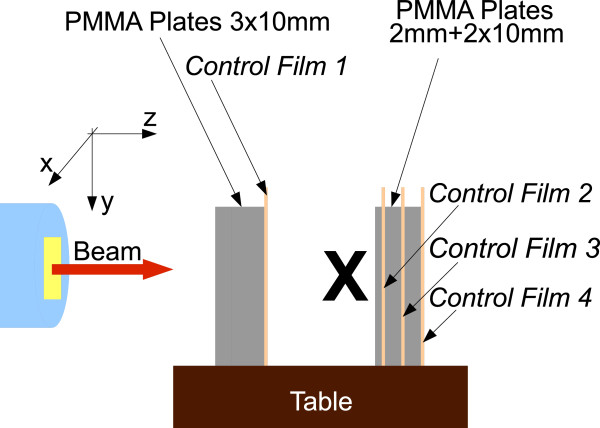
**Schematic drawing of the irradiation setup used for high dose irradiation.** The gel samples were positioned at “**X**“. The control films were used for irradiation verification.

In order to relate the clinical prostate patient irradiation conditions to experimental gel sample irradiation conditions (required due to direct neighbourhood of clinical target volume and investigated material of spacer gel), constant radiobiological effectiveness (RBE) values of 3 for carbon ions and 1.1 for protons were conservatively assumed. These RBE values result mainly from *α*/*β* tissue characteristic ratio applied in treatment planning in the ongoing clinical study on prostate cancer at HIT. Starting from RBE definition:

(2) $$\text{RBE}=\frac{D_{\gamma }}{D_{\text{ion}}}{|}_{\text{Isoeffect}}$$

with the photon absorbed dose *D*_*γ*_ (typically 250 kV X-rays) and the RBE weighted dose *D*_*Ion*_, the carbon ions or protons absorbed (physical) dose was calculated by linking it to the photon absorbed dose (*D*_*γ*_) and used as treatment plan optimisation constraint. The resulting treatment plan specification for both ion species is summarised in Table 1. The gel sample was positioned in the centre of the planned target field. The isocentre of the plan was set in the centre of mass of the target volume cube. The beam was entering along the z direction. The high dose irradiation setup used for all gel samples is schematically reported in Figure 2.

**Table 1:** **Treatment plan specification used for**^**12**^**C and proton high dose irradiation**

	^**12**^**C**	**Protons**
**Prescribed RBE weighted dose**	100 Gy (RBE)	100 Gy (RBE)
**RBE**	3	1.1
**Absorbed target dose**	33 Gy	91 Gy
Target field dimensions(*) X/Y/Z [mm]		
Augmenix sample	25/65/25	25/65/25
Target field dimensions(*) X/Y/Z [mm]		
HIT sample	32/55/40	-
**Beam spot FWHM**	6 mm	12 mm

The target fields in the irradiation plans were designed using safety margins around the target in order to guarantee complete coverage of the sample with the prescribed dose distribution. (*) Dimensions are defined in the beam coordinate system (c.f., Figure 2).

The fully 3D active raster scanning system with feedback from the monitors to the scanning system is used for dose delivery at HIT [20]. During irradiation the therapy control system verified in real-time the lateral position of the beam and the delivered dose according to the prescribed plan. To ensure that the samples were positioned correctly according to the beam line, a set of radiographic films was additionally positioned up- and downstream of the vial.

### PET measurement

#### *Phantom design and experimental setup*

**Figure 3:**
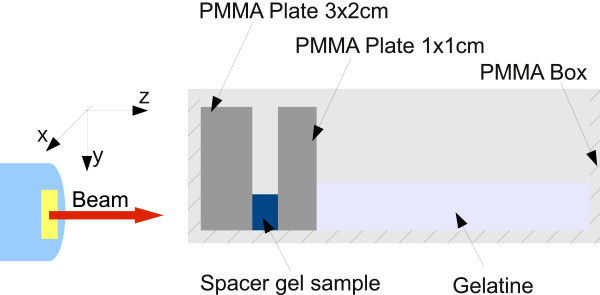
**Schematic drawing of the phantom design used for the PET study.** Several PMMA plates were inserted into a PMMA box, with the spacer gel sample in between. The remaining space downstream the beam direction was filled with gelatin. The phantom was positioned such that the pencil beam entered the geometrical centre of the gel sample.

The phantom was designed in such a way that the already investigated materials, providing a reference medium, were combined with gel samples specifically manufactured with a dedicated geometry. A sketch of the phantom design is presented in Figure 3. A box of (10x10x35) cm^3^ inner dimension made of PMMA plates of 1 cm wall thickness was equipped with several transversely positioned PMMA slabs of various thickness, building a slot where the gel sample was fit in (c.f., Figure 3). The gel insert was positioned in the plateau region of the expected activity depth distribution, i.e. well in front of the distal activity falloff, to reduce uncertainties. The residual volume in depth was filled with gelatin, used as a substitute for water. For the production of the gel sample two dedicated moulds were prepared and sent to Augmenix, where the gel was filled in, resulting in rectangular shaped samples of about (2x4x10) cm^3^. Two identical phantoms were assembled according to the design reported in Figure 3 and irradiated with mono-energetic proton pencil-beams at two different energies, with the beam entering at the PMMA-equipped side. For the irradiation, they were positioned at the centre of overall phantom length and gel height at the laser-indicated iso-centre. After irradiation, the phantoms were transported to the PET/CT device (Siemens Biograph mCT), installed next room and again positioned at the iso-centre. The examination protocol comprises a 30 minute PET acquisition followed by the CT scan of the phantom, which is required for attenuation correction in the PET image reconstruction.

#### *Activity calculation and data analysis*

The expected spatial positron emitter yield is simulated using the Monte Carlo (MC) particle transport and interaction code FLUKA [21,22]. In view of the production of *β*^+^-emitting isotopes, the approach reported in [23] is applied to simulate the yields of ^11^C, ^13^N and ^15^O. In this approach, externally provided production cross sections for the different isotope production channels are coupled to the actual code. The cross-section data sets used in the present study have been validated experimentally at HIT and tuned to model explicitly the proton-irradiation induced activity yield measured with the installed PET device. The material definition of the spacer gel for the simulation was assumed as reported in Section Spacer gel samples. In order to calculate the activity distribution from the simulated positron emitter yield, the detailed time structure of the irradiation and the subsequent time delay to the 30 min PET acquisition is considered as described in (Bauer J, Unholtz D, Kurz C, Parodi K: An experimental approach to validate the Monte Carlo modelling of offline PET/CT-imaging of positron emitters induced by scanned proton beams, *in preparation*).

PET images are reconstructed from the measured data by an iterative OSEM (ordered subset expectation maximization)-based algorithm provided by the scannersoftware. For the data analysis, the same strategy as described in (Bauer J, Unholtz D, Kurz C, Parodi K: An experimental approach to validate the Monte Carlo modelling of offline PET/CT-imaging of positron emitters induced by scanned proton beams, *in preparation*) is pursued, considering the reconstructed static PET image in comparison to the total simulated activity distribution as well as several dynamically reconstructed PET images representing different time points within the total acquisition time. In the latter case, the initial activity contribution of a single isotope fraction at the start of the PET measurement is extracted by fitting the exponential activity decay assuming the presence of the respective isotopes in each material. In order to provide the maximum data statistics, the activity distributions are laterally integrated prior to the fit procedure. As the simulated activity yield depends directly on the material definition, a comparison of the simulated to the experimentally determined isotope fractions allows to verify the chemical material composition.

## Results

### Particle range measurement

Table 2 summarises the obtained results of particle range measurements under reference conditions and in the gel. The results of the reference measurements obtained in measurement session 1, 2 and 3 were normalised on the basis of the range measurement performed without any absorber to calculated value (applied offsets were: 0.00 0.00 mmH_2_O, -0.03 mmH_2_O and 0.69 mmH_2_O, respectively). For the reference measurements in the empty vial and in the vial filled with water (#3-#4) the calculated values of Bragg-Peak position are covered by 2 *σ* field (± 0.2 *mmH*_2_*O*) of experimentally estimated carbon ion depths. The positions of the Bragg-Peak obtained with the vial filled with water and with spacer gel almost overlap. The relative shift is 0.39 ± 0.49 mmH_2_O maximally. The measured mean particle range in the vial filled with gel in all three measurement sets is 38.31 ±0.28 (2*σ*) mmH_2_O, which results in WEPL of 1.011 ±0.011 (2*σ*). As the variation of the Bragg-Peak position for the spacer gel and for water is of the same magnitude within all three measurement sessions, one can conclude that no relevant changes in the range of carbon ions in the gel were observed within 10 weeks period.

**Table 2:** **Calculated [**[24]**] and measured carbon ion ranges (presented in in mm)**

		**Calculated**	**Session 1**	**Session 2**	**Session 3**
#1	**Reference**	86.65	86.65	86.68	85.96
#2	**Phantom-reference**	86.65	86.61	86.69	85.97
#3	**Empty vial**	70.69	70.80	70.80	70.00
#4	**Vial filled with water**	38.51	38.71	38.72	38.02
#5	**Vial filled with gel**	-	[*3*] 38.31 ±0.30	[*5*] 38.27 ±0.29	[*3*] 37.63 ±0.29
#6	**Vial filled with gel**	-	-	-	[*3*] 37.68 ±0.51
	**(After 33 Gy irradiation)**				
#7	**WEPL of spacer gel**	-	1.0126 ±0.0111	1.0141 ±0.0101	1.082 ±0.0108

Sessions 1–3 (columns) refer to three measurement series acquired within 10 weeks. The values in rows #1 to #6 are given in mmH_2_O. Rows #1 to #4 present the ranges acquired in reference measurements (2*σ*=0.2 mmH_2_O), row #5 (2*σ* uncertainty) reports the measured ranges of spacer gel, row #6 (2*σ* uncertainty) the measured range after the high dose irradiation (c.f., Section Insensitivity to high dose irradiation). In squared brackets [*x*] the number of performed measurements is stated. The bottom row (#7) presents calculated WEPL values.

**Figure 4:**
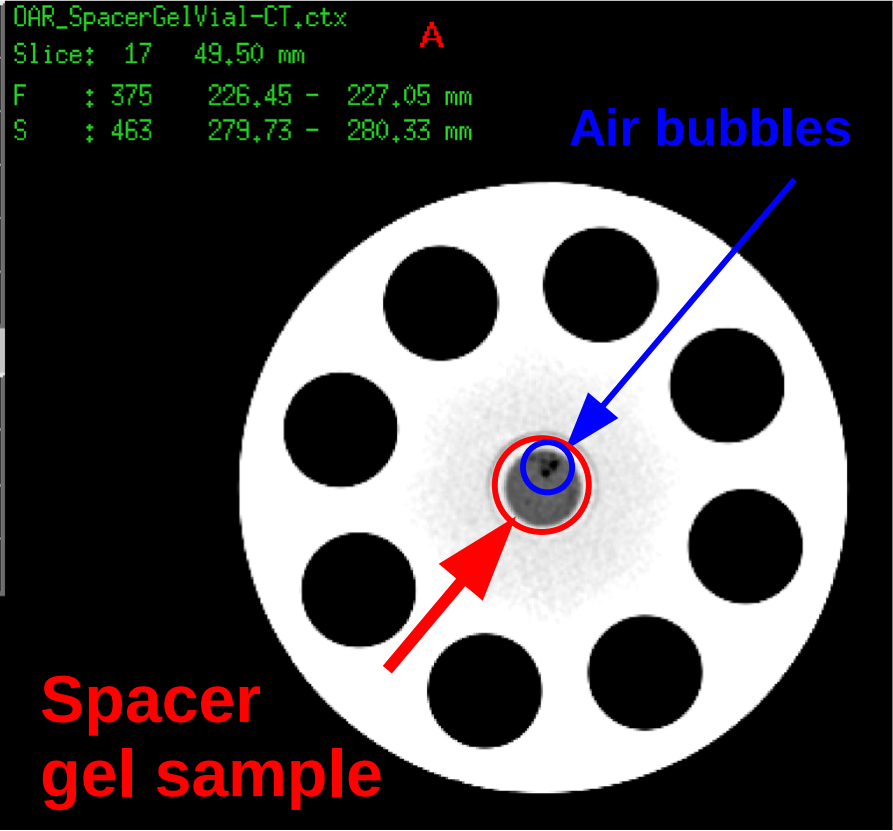
**Transversal view of the spacer gel CT scan used for estimation of the HU value.** Spacer gel was injected to the vial that was positioned in the centre of the phantom.

In order to analyse the compatibility of the spacer gel with the TPS at HIT, the HU value of the spacer gel was determined using HIT-PET/CT by 28.9 ± 6.1 (2*σ*), c.f., Figure 4, which, in accordance with HLUT used at HIT, corresponds to WEPL of 1.027. On the basis of treatment planning CT image, the TPS assumes a WEPL for the particle range calculation insignificantly larger than real particle range during the irradiation. The difference of the measured (real) WEPL of spacer gel to the predicted one (based on CT) is 0.016.

### Insensitivity to high dose irradiation

The spacer gel insensitivity to high dose irradiation was independently investigated by Augmenix and at HIT. The analysis performed at HIT indicated that no particle range changes could be observed due to the exposure of the sample to the absorbed dose of 33 Gy (carbon ions), and 91 Gy (protons). These doses are comparable to 100 Gy (RBE) in target area of prostate patient. The measured mean particle range in the vial filled with gel after the high dose irradiation was 38.37 ±0.51 (2*σ*) mmH_2_O and thus differs from the mean value determined prior to the high dose exposure by 0.06 mmH_2_O (c.f., Table 2, session 3, measurements #5 and #6). The difference in mean value is insignificant with regard to measurement uncertainties, which indicates that the larger standard deviation calculated for peak position measurement obtained after the high dose irradiation in comparison to the previous ones might have been caused by positioning uncertainties.

### PET Measurement

The two PET phantoms containing the spacer gel (c.f., Figure 3) were irradiated with mono-energetic pencil-like proton beams of *E* = 125.67 MeV/u and *E* = 176.75 MeV/u, which reflect a medium ∼12 cm and ∼21 cm deep (penetration depth in water) and were already used in previous measurements in PMMA and gelatin material (Bauer J, Unholtz D, Kurz C, Parodi K: An experimental approach to validate the Monte Carlo modelling of offline PET/CT-imaging of positron emitters induced by scanned proton beams, *in preparation*). As only the higher energetic beam reached the gelatin volume, we concentrate here on the results from this irradiation. The analysis of the low-energy irradiation data yielded a phantom activation that was comparable to the one with the higher energy but was restricted to the PMMA and gel sample regions.

**Figure 5:**
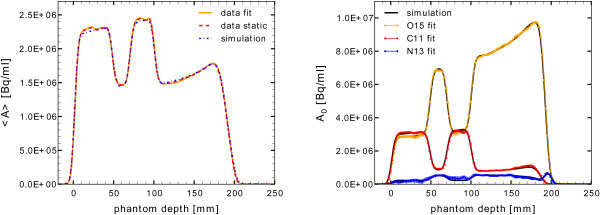
**Activity depth profiles as area normalised distributions.** Left: laterally integrated depth profiles of the activity averaged over the complete measurement frame (<*A*>) as obtained from the static data reconstruction (red, dashed), from the MC simulation (blue, dash-dotted), and the sum of the fitted single isotope contributions (yellow, solid). Right: depth profiles of the initial activity at the PET measurement start (*A*_0_) as extracted from the fit of the dynamically reconstructed PET data, compared to the simulated isotope yields (black lines), for ^15^O (yellow), ^11^C (red) and ^13^N (blue).

The pencil-beam irradiation of the phantom with a total of 2.24·10^11^ protons took 215 s, while the subsequent phantom transport to the PET/CT scanner caused a time delay of 176 s. Figure 5 (left) reports the laterally integrated activity depth profile, normalised to area. Due to a half-life of about 20 minutes, the decay of ^11^C isotopes dominates the measured signal, resulting in a higher activity level in the PMMA regions than in the gel and gelatin regions which are dominated by the shorter lived ^15^O (*T*_1/2_≈2 min.) playing only a minor role in the reconstructed activity image averaged over the total measurement frame time of 30 min. A good overall agreement is observed between the measurement (c.f., Figure 5, red dashed line) and the simulated expectation (c.f., Figure 5, blue dashed-dotted line), especially with regard to the activation of the gel sample and the beam range in this material of interest.

In order to test the validity of the assumed chemical gel composition, different isotope contributions to the total measured activity are determined by the fit analysis described in Section Activity calculation and data analysis calculation and data analysis. The resulting separated activity depth profiles for ^11^C, ^13^N and ^15^O are presented in Figure 5 (right) as area normalised distributions. Qualitatively, the fitted activity fractions agree very well with the simulated expectations, not only in the reference media PMMA and gelatin but also in the gel sample region thus confirming a realistic material definition used for the simulation. As an additional consistency check the activity distribution averaged over the measurement frame is calculated from the sum of the separately fitted isotope contributions and compared to the distributions obtained from the static data reconstruction and respective simulation in Figure 5 (left).

Both analysis strategies, the static and the dynamic data processing, have proven the validity of the assumed qualitative gel properties in comparison to the activity simulation. The relative activity level observed in the different materials agrees very well to the simulated expectation of the frame averaged activity, which is ^11^C dominated, as well as of the initial activity at the start of the PET measurement, when the signal is dominated by the decay of ^15^O. A slight offset of about 5% is observed between the absolute activity values, which is, however, consistent with the uncertainties of the dose calibration that was conducted more than 12 hours prior to the actual measurement.

## Discussion

### Compatibility with the treatment planning system

During the measurement the water column was shifted in 0.1 mm steps, (*u*_*step*_) which results in Bragg-Peak determination uncertainty of comparable level (*u*_*fit*_). The uncertainty in the manufacture of PMMA vial is 0.05 0.05 mm (*u*_*vial*_). These (*u*_*step*_, *u*_*fit*_, *u*_*vial*_) result in uncertainty in Bragg-Peak position determination within an empty vial or vial filled with water of ± 0.2 mmH_2_O (2 *σ*). The uncertainty of sample positioning was compensated by application of offsets between the measurement sessions. The gel injected to the vial did not polymerise not in a homogeneous way but with some air bubbles enclosed in the material (c.f., Figure 4), which happens also when the material is implanted to the patient. This inhomogeneity in the gel sample was partially observed as a variation of the measured particle range after rotating the vial in the vial holder, which is described by standard deviation of performed measurements (*u*_*sample*_).

To consider the fraction of air within the sample filled with the spacer gel, one can assume that 3% of vial volume was filled with air, which corresponds to 1 mm shift of Bragg-Peak in water and to WEPL change of 0.04. Apart from the prior error analyses, air fraction in the sample might cause a significant (in comparison with the other errors) systematic inaccuracy in range measurement. However, this misalignment in the calculation of WEPL of spacer gel material might cause a desired convergence of measured WEPL point with the HLUT.

The range of carbon ion beam in the gel is not exactly equal to the one assumed by the TPS on the basis of the CT image. The error in range calculation occurring during the plan optimisation process is equal to the difference in WEPL (0.017) between the measured (real) WEPL (1.011 ±0.011) of the gel and the WEPL corresponding to the measured HU value of the gel (1.028 WEPL for HU = 28.9). Assuming a typical particle beam penetration depth of 40 mm in the homogeneous gel material, the estimated dose distribution shift that may occur comes up to 0.65 mm for the distal slices of a treatment plan. During the treatment planning process the clinical target volume (CTV) is extended to the planning target volume (PTV) in order to cover uncertainties of the treatment delivery technique. At HIT, a PTV safety margin of 5 to 7 mm is normally applied in the pelvic area. Dealing with the error resulting from an application of spacer gel as a random error (not the whole spacer gel implant is in the dose field, the amount of the gel in the dose field depends on random daily anatomy and positioning variations) we can add it to the existing margins by calculating the root square of the sum of the squares of possible dose shifts induced by spacer gel and by other therapy uncertainties. This consideration results in a maximal margin extension of 0.1 mm. As this uncertainty affects the prostate patient irradiation marginally, it could be neglected for the creation of the PTV.

### Insensitivity to high dose irradiation

During sample irradiation lateral position of the beam and dose deposited to the samples were monitored according to the prescribed plan by the monitors of raster scan system, as it is performed during the patient irradiation. The irradiation field larger than the irradiated target, exact laser positioning system and control radiographic films guaranteed deposition of the prescribed dose in the samples.

After an exposure to 150 Gy photon irradiation, and to 33 Gy carbon ion and 91 Gy proton irradiation Augmenix performed functional and chemical tests. The tests included an evaluation of hydrogel swelling and time to complete hydrolysis in accelerated conditions. Additionally, following hydrolysis, solutions were evaluated with Nuclear Magnetic Resonance (NMR) spectrometry providing the information about the distribution of chemical species contained in a sample and Gel Permeation Chromatography (GPC) which is a chromatography method typically used for analysis of molecular weight of polymers.

Augmenix reported that the high dose irradiation did not impact the hydrogel swelling or hydrolysis rate, demonstrating that the hydrogel network was not functionally altered. Additionally, NMR and GPC analysis performed on complete hydrolysed samples found no differences in the irradiated and non-irradiated hydrogels, suggesting that this level of irradiation does not result in significant (in comparison with control sample) hydrogel molecular rearrangement.

### Role for PET-based treatment monitoring

The presented measurement of the proton-induced *β*^+^-activity in the spacer gel sample confirms the assumed material modelling. We were able to verify experimentally the composition relevant for PET imaging by analysing the produced positron emitter yield separated by isotope.

As the gel consists mainly of water, the activity induced by the proton beam is dominated by the decay of ^15^O. Due to the comparatively short half-life of this isotope of about 2 minutes, most of this signal is lost during the patient transfer from the treatment place to the PET/CT device and necessary patient repositioning at the scanner, which usually takes about 5–10 minutes, depending on the complexity of the immobilisation equipment and the patient’s condition. Therefore, the residual activity in the spacer gel that can be detected in the offline PET-measurement is expected to be rather low compared to the activity level in the surrounding tissue, where more ^11^C is produced. However, as the spacer gel is a confined object delimited from the surrounding perfused tissue, the induced activity remains at the production spot and is not affected by the biological washout of the induced activity, which reduces significantly the measurable signal in well-perfused human tissue region. Considering a typical prostate proton-therapy fraction with a single RBE weighted dose of 3.3 Gy (RBE), the activity level detected in the spacer gel area is about 300 Bq/ml, which is similar to the signal observed in the surrounding low-perfused bony and fatty structures, where washout plays only a minor role. In case of carbon ion irradiation, the PET image obtained offline is much less sensitive to the target activation, since the signal is dominated by the ^11^C projectile fragments. Thus, the distal regions of the treatment field exhibit a pronounced signal enhancement with respect to the dose plateau region. As the spacer gel is typically located at the edge of the distal dose fall-off region of the single fields, the activation of the gel is amplified by the projectile fragments stopped inside the gel.

The performed investigations show that spacer gel irradiated with proton and carbon ion induces a signal which can be obtained using the post-therapeutic PET-imaging. Assuming stable position of the gel between prostate and rectum over the whole therapy course, the irradiation-induced activity within the spacer gel provides additional information useful for validation of treatment delivery with post-therapeutic PET-imaging for both proton and carbon ion irradiation. The additional reference and marker application of spacer gel would certainly benefit from a higher carbon fraction in the material, such that more ^11^C is generated, and from an optimised treatment time course reducing the irradiation and treatment time course.

### Clinical aspects

The clinical and dosimetric advantages of the application of spacer gel for photon therapy were already discussed by Pinkawa et al. [11]. These are mainly a reduced dose to the rectum and the potential for application of more rigorous treatment planning objectives. The investigations presented in this paper show that the physical changes in the spacer gel material during the therapy course negligibly influence the irradiation of the prostate target. Since spacer is a soft implant of tissue-like density and gel-consistency, there are justifiable presumptions that spacer gel may deform during the therapy course. Pinkawa et al. [25] reports that the distance between the prostate and anterior rectal wall is stable when spacer gel is implanted before treatment planning. The ion therapy is particularly sensitive on the anatomy motion and deformations within the target and in the beam entrance path. These effects cause range variations and significantly influence the quality of irradiation [26]. It is expected that the range variations caused by the motion of the target, possible deformation of the spacer gel, or density variations in the beam entrance path might influence the quality of irradiation of prostate patient to a greater extent than the uncertainties caused by implantation of the spacer gel material. The quantitative analysis of dosimetric effects of the application of spacer gel in particle therapy in the presence of anatomical variations in the target region is one of the main topics of ongoing investigations at HIT in this field. The results of measurements described in this document were a basis for the application of spacer gel in the clinical study “Ion Prostate Irradiation” ( http://www.clinicaltrials.gov/) currently performed at HIT (2012).

The application of the spacer gel would have an additional advantage if the gel had radio-opaque properties. In radiation therapy gold markers implanted to the tissue (i.e., prostate) are used to visualise the target by pre-treatment control radiography or CT imaging. In the same way, spacer gel with radio-opaque properties, located in direct neighbourhood of the prostate, might be used to indicate the position of the target or at least to recognise these treatment fraction, when anatomy misalignments (filling of rectum or bladder different than during treatment planning) would cause unacceptable decrease of irradiation quality. Such products were recently commissioned for clinical application by Augmenix in US.

## Conclusions

The performed investigations show that the range of carbon ions in spacer gel remain stable over the time interval of ten weeks and under the exposure of a high dose of 33 Gy (carbon ions), and 91 Gy (protons), which is comparable to 100 Gy (RBE) in patient. The spacer gel reveals no physical changes after therapeutic doses of carbon ion or proton irradiation. The penetration depth of these ions is properly predicted by the treatment planning system using computed tomography imaging that allows its safe usage during the particle therapy of prostate cancer. The application of the spacer gel implant in the close neighbourhood of prostate is expected to allow higher doses to the clinical target volume and to spare the most critical organ at risk. Further research on the composition of spacer gel material might lead to additional clinical benefits by a validation of prostate patient treatment via PET-imaging or by patient positioning based on the gel as a radio-opaque marker. Generally, the usage of soft implants enlarging the space between the organs, not being involved in biological processes and having the function of a marker, could provide benefits for several clinical aspects of radiation and particle therapy.

## Abbreviations

CT: Computed tomography; CTV: Clinical target volume; DRR: Digital X-ray radiography; FOV: Field of view; FWHM: Full width at half maximum; HU: Hounsfield unit; HIT: Heidelberg Ion Beam Therapy Centre; HLUT: Hounsfield unit look up tables; IMRT: Intensity modulated radiation therapy; MC: Monte Carlo; NMR: Nuclear magnetic resonance; OAR: Organs at risk; OSEM: Ordered subset expectation maximization; PEG: Poly-ethylene glycol; PET: Positron emission tomography; PMMA: Poly-methyl methacrylate; PTV: Clinical target volume; RBE: Radio-biological effectiveness; GPC: Gel Permeation chromatography; TPS: Treatment planning system; TRiP98: Treatment panning for particles (software package).

## Competing interests

The authors declare that they have no competing interests.

## Authors’ contributions

AR under the supervision of SB and TH designed and performed the experiments with carbon ion beams at HIT. JB and AR under the supervision of KP designed and performed PET experiments. The analysis of dynamically reconstructed PET data was feasible thanks to the software package developed by DU. PC provided the spacer gel samples, supervised and reported the experiments performed by Augmenix. The clinical aspects were consulted by GH, KH and JD. AR and JB drafted the manuscript. KP, CB, OJ and TH reviewed the text of the manuscript. All authors read and approved the final manuscript.
